# Treatment of femoral neck fractures in elderly patients over 60 years of age - which is the ideal modality of primary joint replacement?

**DOI:** 10.1186/1754-9493-4-16

**Published:** 2010-10-20

**Authors:** Christian Ossendorf, Max J Scheyerer, Guido A Wanner, Hans-Peter Simmen, Clément ML Werner

**Affiliations:** 1 Department of Surgery, Division of Trauma Surgery, University of Zurich, Raemistrasse 100, 8091 Zurich, Switzerland

## Abstract

**Background:**

Femoral neck fractures in the elderly are frequent, represent a great health care problem, and have a significant impact on health insurance costs. Reconstruction options using hip arthroplasty include unipolar or bipolar hemiarthroplasty (HA), and total hip arthroplasty (THA). The purpose of this review is to discuss the indications, limitations, and pitfalls of each of these techniques.

**Methods:**

The Pubmed database was searched for all articles on femoral neck fracture and for the reconstruction options presented in this review using the search terms "femoral neck fracture", "unipolar hemiarthroplasty", "bipolar hemiarthroplasty", and "total hip arthroplasty". In addition, cross-referencing was used to cover articles eventually undetected by the respective search strategies. The resulting articles were then reviewed with regard to the different techniques, outcome and complications of the distinct reconstruction options.

**Results:**

THA yields the best functional results in patients with displaced femoral neck fractures with complication rates comparable to HA. THA is beneficially implanted using an anterior approach exploiting the internervous plane between the tensor fasciae latae and the sartorius muscles allowing for immediate full weight-bearing. Based on our findings, bipolar hemiarthroplasty, similar to unipolar hemiarthroplasty, cannot restorate neither anatomical nor biomechanical features of the hip joint. Therefore, it can only be recommended as a second line of defense-procedure for patients with low functional demands and limited live expectancy.

**Conclusions:**

THA is the treatment of choice for femoral neck fractures in patients older than 60 years. HA should only be implanted in patients with limited life expectancy.

## Background

Femoral neck fractures are frequent injuries in the patient population of every trauma center and have a high incidence in the general population. Paralleling trends of demographic forecasts, their incidence will continue to rise in the future [1,2]. Especially in the elderly, femoral neck fractures represent a significant health care problem and have enormous impact on health insurance costs. Therefore, the appropriate treatment of femoral neck fractures is mandatory. Today, surgery is the mainstay of care. While in younger patients (20-50 years), closed reduction and internal fixation (CRIF) is routinely performed, the treatment of older patients with intracapsular femoral neck fractures largely depends on local conditions, patient profiles, personal preferences and training of the surgeon. This is merely based on personal believes determining the management of patients than evidence from the literature [3,4]. In the authors' country e.g. general surgeons provide care in musculoskeletal trauma, while this care is provided by orthopaedic trauma surgeons elsewhere. Often, the type of implant is dictated by the surgeons training. Worldwide, some surgeons treat older patients similar to younger ones by CRIF using cannulated screws or devices like the sliding hip screw. In contrast, reconstruction options include: hemiarthroplasty (HA) - unipolar and bipolar - and total hip arthroplasty (THA). Hence, the optimal treatment of this patient population is still under debate [5]. Therefore, surgeons cannot be sure, whether they offer the best care available to their patients. The present review focuses on the treatment of femoral neck fractures in patients of 60 years and older to discuss indications, techniques, limitations and problems of each of these techniques. It aims to distil from the literature the best available treatment for this important patient population.

## Methods

We performed a review of all studies about femoral neck fractures treated with either hemiarthroplasty or THA published between Jan 01, 1975 and December 31, 2009. All publications were derived from NCBI-PubMed http://www.pubmed.gov using the search-term "femoral neck fracture" combined with "unipolar hemiarthroplasty", "bipolar hemiarthroplasty", or "total hip arthroplasty", and "total hip replacement", respectively. Studies other than written in English, French, Spanish or German were disregarded. Those about non-traumatic etiologies, e.g. degenerative joint disease, revision arthroplasty, pathologic fractures or rheumatoid arthritis, articles covering hip resurfacing, and biomechanical studies, as well as case reports were excluded from the present study. Duplicates were eliminated from further analysis.

## Results

### Hemiarthroplasty (Uni- and bipolar)

**Figure 1:**
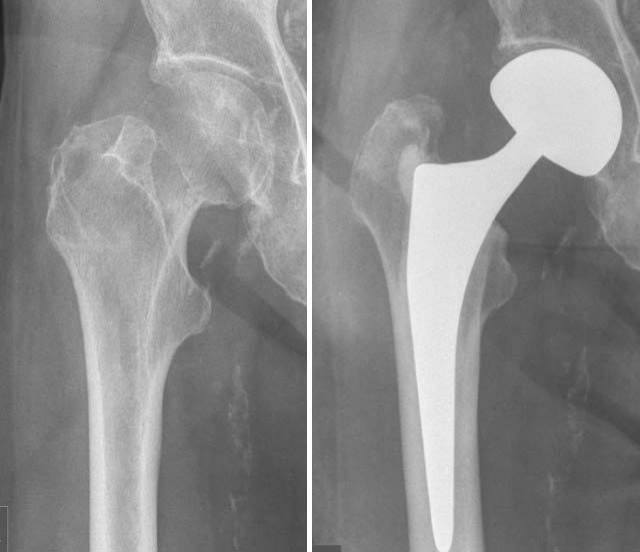
**Monopolar hemiarthroplasty. Femoral neck fracture treated with unipolar hemiathroplasty**.

**Figure 2:**
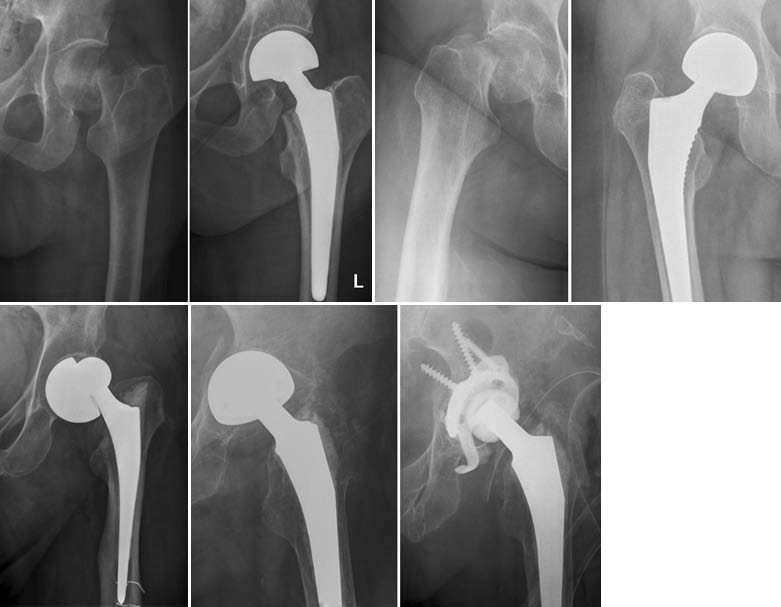
**Femoral neck fracture treated with bipolar hemiarthroplasty. **Cemented (upper left panel) and press fit technique (upper right panel). Complications of bipolar hemiarthroplasty include luxation (lower left panel) and protrusion of the acetabulum, her treated by total hip arthroplasty (lower right panel).

Advantages of monopolar (Figure 1) and bipolar arthroplasty (Figure 2) compared to THA include short operation time and quick mobilization of the patient. Good or at least acceptable clinical, functional and radiological results have been reported in a wide array of studies [6-20]. However, whether unipolar or bipolar hemiarthroplasty (HA) provide better results is still under debate [20-24].

Single component devices are based on models pioneered by Moore and Thompson in the 1950's [21,22]. Since then, this type of prosthesis has gained popularity for the treatment of displaced femoral neck fractures because of its ease to implant in a short period of time. In addition, the Bateman prosthesis, designed by Bateman in the early 1970s to reduce acetabular wear as frequently seen with the Moore and Thompson types, was an important innovation with better functional outcome assessed by the Harris hip score [23,24]. Monopolar HA cause significant acetabular wear and subsequent problems [25]. Reduction usually is somewhat more difficult in monopolar arthroplasty compared to bipolar ones and particularly to THA.

Bipolar hemiprostheses consists of a metal cup that serves as an outer head, a metal femoral component, and a polyethylene insert in between. Here, a multiple-bearing principle is effected by creating a double layer of universal motion. The major movement occurs at the inner bearing, as the addition of weight shifts most of the motion to the inner bearing reducing the damaging effect of metal against the acetabular floor. That way, a low-friction layer at the metal head-plastic interface, with much less frictional torque than the one developed at the outer shell acetabular interface is provided [26]. Usually, these prosthetic devices are implanted using a lateral Hardinge, i.e. transgluteal or an anterolateral Watson-Jones, approach [27-29]. Both approaches provide good visualization of acetabulum and femur while somewhat decreasing the risk of luxation compared to a posterior approach. The disadvantages and dangers of implanting arthroplasty using a transgluteal approach have been extensively documented, including malorientation of the cup, destruction of the abductor mechanism by cutting the external rotators making immediate full weight-bearing is impossible [30-35].

Another disadvantage of bipolar hemiarthroplasty lies within the construction principle: after approximately one year, bipolar hemiarthroplasty act as unipolar hemiarthroplasty [36-38], as the outer metal-on-bone friction is supposed to be higher and movements transferred to the inner metal-on-polyethylene bearing in arthritic patients [38]. Consequently the bipolar cup tend to horizontalize and remain within this position (Figure 2), [38-43]. However, the exact clinical consequences of this unintended positioning remain unclear.

There is a considerable complication rate in unipolar and bipolar hemiarthroplasty [44], independent of whether a junior or senior surgeon performed the procedure [45]. Hemiarthroplasty comprise considerable need for reoperation often necessitating conversion to THA [46-48]. This may also be explained by neglecting - and not restoring - the femoral offset [49,50]. due to old-fashioned design which does account for anatomical and biomechanical features of the hip joint and the femoral neck. Hence, restoring the individual offset is often impossible using unipolar and bipolar hemiarthroplasty. Consecutively, sufficient tissue tension balancing is impaired. Adequate treatment of double fond conditions of the acetabulum cannot be acceptably addressed using bipolar HA.

Further complications of bipolar hemiarthroplasty include intra-operative metaphyseal fractures reported in 10% of cases in a series of 273 patients with displaced femoral neck fractures, depending on extension of fracture dislocation [51]. The dislocation rate was reported to be 1.5% in a large series of 1934 hips [52], half of which redislocate after reduction. Other authors reported 4% dislocation rate [53,54]. Additional problems associated with bipolar hemiarthroplasty are migration of the bipolar head, as well as stem migration [55], failure of the polyethylene inlay [56], and component disassembly [57-61]. Heterotopic ossification is more frequent in cemented than in uncemented bipolar hemiarthroplasty [62]. However, pain relief and function are better in patients with bipolar hemiarthroplasty -regardless of cemented or uncemented- compared to unipolar arthroplasty [63]. In order not to miss anterior narrowing of the joint line, axial views must be done as cross-table.

Both, unipolar and bipolar hemiarthroplasty increase biomechanical stresses on the acetabular bone and that way cause migration of the head with consecutive destruction of the acetabulum, as demonstrated in a finite element model [64]. Although some reports describe little acetabular erosion [65-67], several studies demonstrated significant acetabular wear in up to 67% of cases, resulting in an average time to failure of 38 months [8,68,69]. This wear was quantified with an average rate of 0.7 mm per year [68]. These prostheses are therefore only recommended in old patients with limited life expectancy. A recent review of the current evidence for internal fixation versus hemiarthroplasty versus primary total hip arthroplasty for displaced femoral neck fractures showed no difference in mortality, postoperative pain, function, or quality of life for either of the devices. For hemiarthroplasty, the data suggest minimal differences in outcome between the prosthesis types [70]. Summing up the findings from the literature, hemiarthroplasty are preferentially performed in older patients with limited life expectancy and low functional demands.

### Total Hip Arthroplasty

**Figure 3:**
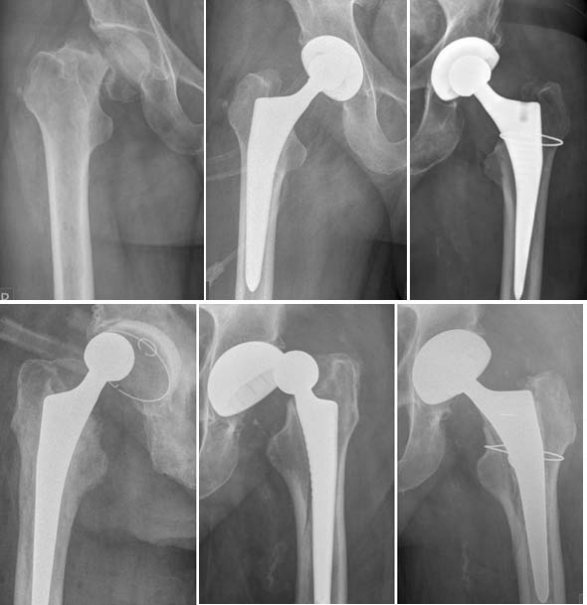
**Femoral neck fracture treated with total hip arthroplasty (upper left panel)**. Potential complications and pitfalls include fracture of the trochanter (upper right panel), luxation (lower left panel), both (lower right panel).

In most western European countries and in the U.S., arthroplasty is the mainstay of surgical treatment of intracapsular femoral neck fractures in patients older than 60 to 65 years [71-73]. Here, total hip arthroplasty yields good clinical short to long-term results [1,74] with significantly less pain and better outcomes represented by quality of life and functional scores [75] (SF-36 and WOMAC) compared to hemiarthroplasty [76]. Along with the improvement of implants, THA has gained attention for the treatment of displaced femoral neck fracture and importance even in countries traditionally treating this group of patients with internal fixation or with unipolar devices (Figure 3).

In a 1993 series, the following prognostic factors were found: nursing home patient, chronic pulmonary disease, serum creatinine level greater than 1.7 mg/100 ml, pneumonia, previous myocardial infarction, duration of surgery, and gender. In contrast, the following factors had no significant influence on mortality: age, time delay from admission to surgery, mode of anesthesia, and cerebrovascular diseases [77]. In contrast, sex, age, waiting time before surgery, stroke, dislocation of the prosthesis and perioperative fracture were identified as key factors negatively influencing outcome at 3 months postoperatively [78].

Independent of the surgical procedure (internal fixation, HA, THA), no statistical effect of time to surgery on mortality could be shown in a series of 2916 patients [79]. In contrast, in a smaller study including 265 consecutive patients, delayed hospitalization of more than 6 hours after trauma was related to higher mortality [80]. However, early surgery within 24 hours was associated with reduced length of hospitalization, although mortality was higher in men at 4 and 12 months with administrative delay in surgery compared to patients with no delay [81,82]. To adequately restore physical activities after surgery, intensive rehabilitation schemes are of paramount importance [83].

Transfusion rates have been shown to be less using minimally invasive techniques [84]. Here, dislocation rates were lower than using other approaches [85-87]. Particularly the posterior approach makes THA prone to dislocation [88]. The anterior approach allows for immediate full weight-bearing, as only the pars reflecta of the rectus femoris muscle is partially detached for this approach and the external rotators of the hip are protected from preventable iatrogenic damage. In contrast, the lateral cutaneous femoral nerve may be at risk using this approach.

Studies comparing bipolar hemiarthroplasty and total hip arthroplasty (THA) showed that THA yields better function at the same complication rate approximately one year postoperatively [89]. Here, functional outcome was better in THA patients than in patients with HA [73,90]. Comparing internal fixation, unipolar Hemiarthroplasty, bipolar hemiarthroplasty and total hip arthroplasty, THA was the most cost effective treatment [91]. In the appropriate patient population, outcomes following total hip arthroplasty are favorable and appear to be superior to those of internal fixation. THA patients had less pain and better function compared to HA patients at the same rate of complications two years postoperatively [76], and bipolar hemiarthroplasty always showed inferior function, worse long-term results, and higher revision rates [46-48,92]. Therefore, conversion of HA to THA is necessary at times [68,93]. In comparison to internal fixation alone, arthroplasty appears superior and significantly reduces the number of surgical revisions. It decreases the rate of complications without increased mortality [1,5]. After 2 years of follow-up, the rate of secondary fracture dislocation and need for revision surgery was less in patients treated with THA compared to those treated with cannulated screws [37].

After failed internal fixation, conversion to THA can effectively restore function and relieve pain, too [94]. As the costs of revision arthroplasty are immense and the patients are even older at this point of time, physically fit patients may beneficially be treated with primary THA [95].

THA is not suitable for every patient including multimorbid patients, or patients with limited live expectancy [96]. Migration at two years is is predictive of the long-term evolution of an implant; cup migration of 1 mm or more at 2 years is predictive of late failure [97].

Disadvantages of THA include higher blood loss and costs compared to bipolar hemiarthroplasty which are approximately $12'290 vs. to $8876 for monopolar hemiarthroplasty [63]. Although the initial costs of THA are greater compared to unipolar or bipolar devices, overall costs are regarded as lower, because of increased long-term survival, better outcome and less frequent reoperations. Complications of femoral neck fracture comprise femoral implant failure including the stem, neck, and the modular head-neck junction, and loosening. Loosening is primarily associated with wear particles [98,99] 0.1 mm wear per year is the threshold for a normal wear rate [100]. Kaplan Meier gives a probability of 94% of keeping the prosthesis for 8 years [101]. Poor treatment results in hemiarthroplasty were observed regardless of them being cemented or not due to failure to restorate neck length and offset [102-105].

The rate of dislocation of THA was reported between in up to 15% of using standard approaches to the hip joint [106]. R rate of revision was higher in patients treated for fractures than in those treated for other reasons with dislocation and periprosthetic fracture being the most frequent causes of revision [38,107,108], as usual 10-year survivorship is around 99% [109].

Initial costs of THA are higher compared to unipolar or bipolar hemiarthroplasty. However, overall costs, including those for revision surgery are lower the outcome of THA is better. In conclusion, THA is recommended as an evidence-based primary treatment for femoral neck fractures in the elderly.

## Conclusions

Total hip arthroplasty is the treatment of choice for femoral neck fractures in patients older than 60 years. Bipolar hemiarthroplasty were shown to be of limited value. Monopolar hemiarthroplasty should only be implanted in patients with limited life expectancy and/or very low functional demands. Total hip arthroplasty ought to be performed by a qualified surgeon in a decent environment of a medium to large trauma or orthopaedic center. Using an anterior approach to the hip enables the patient to immediate full weight bearing.

## Competing interests

The authors declare that they have no competing interests.

## Authors' contributions

CO carried out the literature research and drafted the manuscript. MJS carried out the literature research and participated in drafting the manuscript. GAW and HPS participated in the design of the study and helped to draft the manuscript. CMLW conceived the study, participated in its design drafted the manuscript. All authors read and approved the final manuscript.
